# Minimal Change in the Cytoplasmic Calcium Dynamics in Striatal GABAergic Neurons of a DYT1 Dystonia Knock-In Mouse Model 

**DOI:** 10.1371/journal.pone.0080793

**Published:** 2013-11-19

**Authors:** Sadahiro Iwabuchi, Jin-Young Koh, Kai Wang, K. W. David Ho, N. Charles Harata

**Affiliations:** 1 Department of Molecular Physiology and Biophysics, University of Iowa Carver College of Medicine, Iowa City, Iowa, United States of America; 2 Department of Biostatistics, University of Iowa, College of Public Health, Iowa City, Iowa, United States of America; 3 Medical Scientist Training Program, University of Iowa Carver College of Medicine, Iowa City, Iowa, United States of America; University of California, Berkeley, United States of America

## Abstract

DYT1 dystonia is the most common hereditary form of primary torsion dystonia. This autosomal-dominant disorder is characterized by involuntary muscle contractions that cause sustained twisting and repetitive movements. It is caused by an in-frame deletion in the *TOR1A* gene, leading to the deletion of a glutamic acid residue in the torsinA protein. Heterozygous knock-in mice, which reproduce the genetic mutation in human patients, have abnormalities in synaptic transmission at the principal GABAergic neurons in the striatum, a brain structure that is involved in the execution and modulation of motor activity. However, whether this mutation affects the excitability of striatal GABAergic neurons has not been investigated in this animal model. Here, we examined the excitability of cultured striatal neurons obtained from heterozygous knock-in mice, using calcium imaging as indirect readout. Immunofluorescence revealed that more than 97% of these neurons are positive for a marker of GABAergic neurons, and that more than 92% are also positive for a marker of medium spiny neurons, indicating that these are mixed cultures of mostly medium spiny neurons and a few (~5%) GABAergic interneurons. When these neurons were depolarized by field stimulation, the calcium concentration in the dendrites increased rapidly and then decayed slowly. The amplitudes of calcium transients were larger in heterozygous neurons than in wild-type neurons, resulting in ~15% increase in cumulative calcium transients during a train of stimuli. However, there was no change in other parameters of calcium dynamics. Given that calcium dynamics reflect neuronal excitability, these results suggest that the mutation only slightly increases the excitability of striatal GABAergic neurons in DYT1 dystonia.

## Introduction

The majority of neurons in the striatum release the inhibitory neurotransmitter γ-aminobutyric acid (GABA), and the activity of these GABAergic neurons plays an important role in the motor control of vertebrate animals [1-3]. Dysregulation of these neurons can contribute to the abnormal coordination of network functions and can lead to neurological movement disorders, including Huntington’s disease, Parkinson’s disease, and dystonia [3]. Striatal GABAergic neurons include medium spiny neurons (representing ~95% of all striatal neurons), which project to the substantia nigra pars reticulata and the external segment of the globus pallidus [1,4-6], and GABAergic interneurons (representing ~4% of the striatal neurons), which drive feed-forward inhibition to the projecting medium spiny neurons [5,7,8]. The output of these GABAergic neurons is controlled by both synaptic input and their intrinsic neuronal excitability. 

Synaptic abnormalities have been reported in the striatum of animal models of DYT1 dystonia, the most common hereditary form of movement disorder dystonia, a neurological syndrome that is characterized by involuntary muscle contractions that cause sustained twisting and repetitive movements [9]. The inheritance pattern of DYT1 dystonia is autosomal dominant, and the genetic defect is an in-frame deletion of three nucleotides in the coding region of the *TOR1A* (*DYT1*) gene, leading to the loss of a single glutamic acid residue in the resulting protein, torsinA. TorsinA is an evolutionarily conserved protein that belongs to the AAA+ superfamily [10,11] and is expressed throughout the central nervous system [12], where it likely plays a key role. However, the precise function of this protein—and the mutated form that causes DYT1 dystonia—remains poorly understood. Using heterozygous ΔE-torsinA knock-in mice that harbor the orthologous mutation as in human patients (i.e., patients are heterozygous for the ΔE-torsinA allele) [13,14], several abnormalities in synaptic transmission within the striatum were recently reported. For example, whereas wild-type neurons have suppressed glutamate-mediated excitatory synaptic transmission at cortico-striatal synapses following high-frequency stimulation (i.e., long-term depression was induced), heterozygous neurons did not exhibit this suppression when subjected to the same high-frequency stimulation [15]. In addition, glutamate-mediated transmission at thalamo-striatal synapses is usually followed by a period of suppressed neuronal firing due to activation of dopamine D2 receptors, and this period of suppressed activity was shorter in the heterozygous neurons than in wild-type neurons [16]. Finally, the release of dopamine in the striatum was suppressed in the heterozygous mice relative to wild-type mice [17]. 

Unlike the aforementioned perturbations in synaptic regulation, whether the excitability of striatal GABAergic neurons is altered in the ΔE-torsinA knock-in mouse has not been investigated. Although a recent study found no change in the excitability of striatal cholinergic interneurons [16], which comprise ~1% of striatal neurons and contribute to the regulation and plasticity of synaptic transmission [18], excitability has not been studied specifically in GABAergic neurons, which comprise the majority of striatal neurons.

Here, we measured the excitability of striatal GABAergic neurons cultured from wild-type and heterozygous ΔE-torsinA knock-in mice, using fluorescence imaging of cytoplasmic Ca^2+^ concentration ([Ca^2+^]_c_). A change in [Ca^2+^]_c_ reflects neuronal excitability [19] and was induced by subjecting the neurons to depolarizing electrical stimuli. We found that the amplitude of the stimulus-induced [Ca^2+^]_c_ transients within the dendrites was slightly larger in heterozygous than in wild-type neurons. Given that our immunocytochemical analysis revealed that the majority of cultured striatal neurons are GABAergic, these data show that the ΔE-torsinA mutation increases the [Ca^2+^]_c_ dynamics of striatal GABAergic neurons, indicating a similar, slight increase in intrinsic excitability. 

## Results

### Amplitude and decay time course of [Ca^2+^]_c_ transients in response to a single stimulus

**Figure 1:**
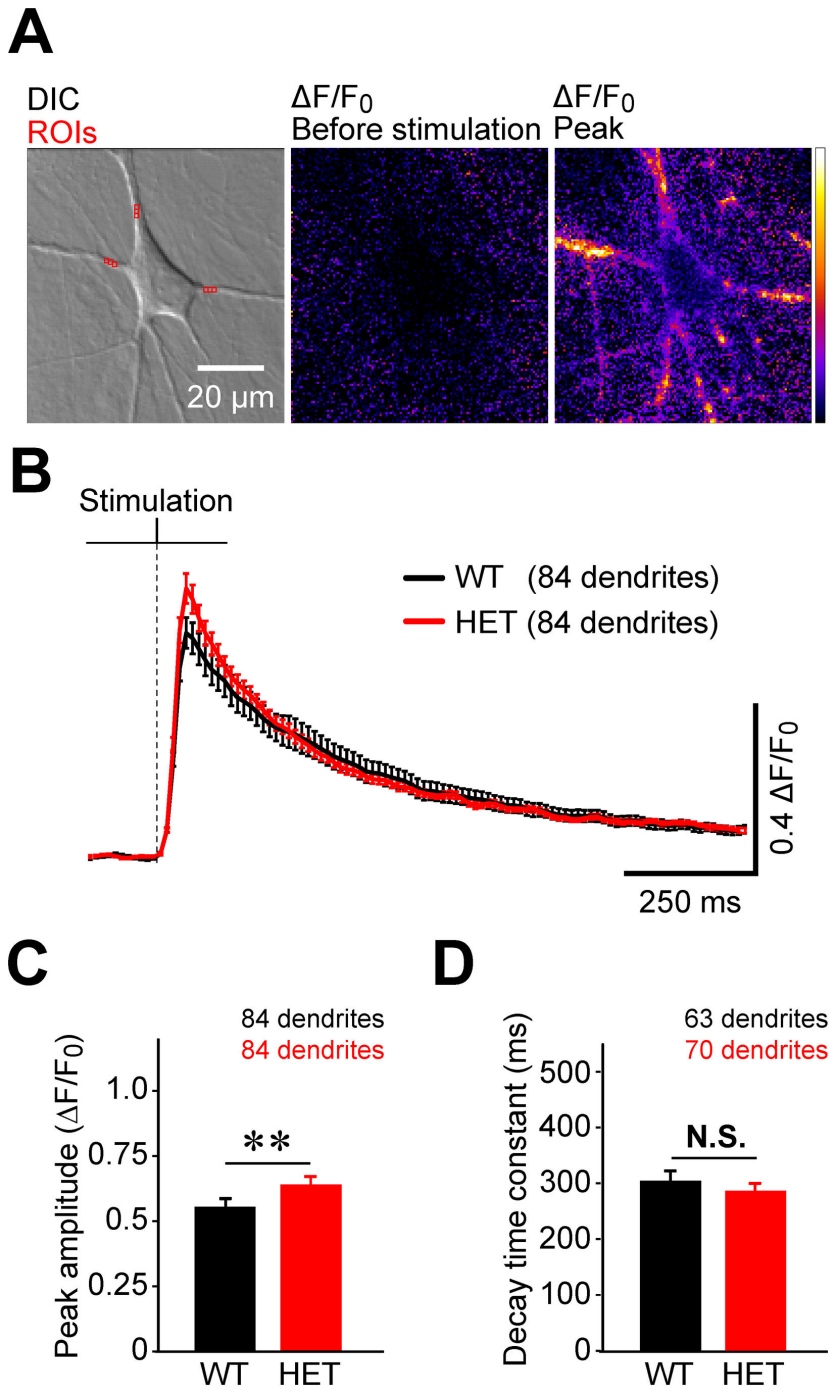
Stimulus-induced Ca^2+^ transients are slightly enhanced by the ΔE-torsinA mutation. (A) Left panel: A cultured striatal neuron obtained from a wild-type mouse imaged using differential interference contrast optics (DIC). Three regions of interests (ROIs) that were used for measuring fluorescence intensity are shown in red. Middle and right panels: The change in cytoplasmic Ca^2+^ concentration ([Ca^2+^]_c_) is expressed as the fold increase in the fluorescence intensity of the Ca^2+^ dye Fluo-5F from the baseline (ΔF/F_0_), before stimulation (middle panel) and at the time of peak intensity (right panel). (B) Average time course of ΔF/F_0_ in wild-type (WT, black) and heterozygous ΔE-torsinA knock-in (HET, red) neurons. The stimulus timing is indicated. (C) There was a difference in the peak amplitude of the response between wild-type and heterozygous neurons (**, p<0.01). (D) There was no difference in the decay time constant between wild-type and heterozygous neurons (p>0.6). N.S., not significant.

We evaluated six properties of the [Ca^2+^]_c_ transients in striatal neurons cultured from heterozygous ΔE-torsinA knock-in mice and wild-type littermates. First, we measured the [Ca^2+^]_c_ dynamics when we depolarized the cultured neurons with a single electrical stimulus delivered by passing a 1-msec current pulse through the bath solution (Figure 1). A differential interference contrast (DIC) image of a representative wild-type neuron loaded with the calcium indicator Fluo-5F-AM is shown in Figure 1A (left panel). Application of an electrical stimulus triggered an increase in fluorescence intensity that corresponded with an increase in [Ca^2+^]_c_. The fluorescence signals were expressed as fold increase relative to the baseline level (ΔF/F_0_; Figure 1A, middle and right panels). The time course of the change in [Ca^2+^]_c_ was plotted by measuring the averaged ΔF/F_0_ values of regions-of-interest in the dendrites (the regions-of-interest, ROIs, are shown in red in the DIC image in Figure 1A, left). The ROIs were placed in proximal dendrites at a fixed distance (7 μm) from the soma-dendrite border. The [Ca^2+^]_c_ increased synchronously in response to the stimulus, reached its peak within a few tens of milliseconds, and then decayed slowly (Figure 1B). 

The peak amplitudes were larger in the heterozygous knock-in neurons (red) than in wild-type neurons (black) (p<0.01, Figure 1C). The time course of the decay was analyzed by comparing the decay time constants, which were measured by fitting single-exponential functions to the decay phases. In contrast to the peak amplitude, no difference was detected between heterozygous and wild-type neurons (p>0.6, Figure 1D). Overall, the responses to a single electrical field stimulus were larger in heterozygous than in wild-type neurons. 

### Induction mechanisms of [Ca^2+^]_c_ transients

**Figure 2:**
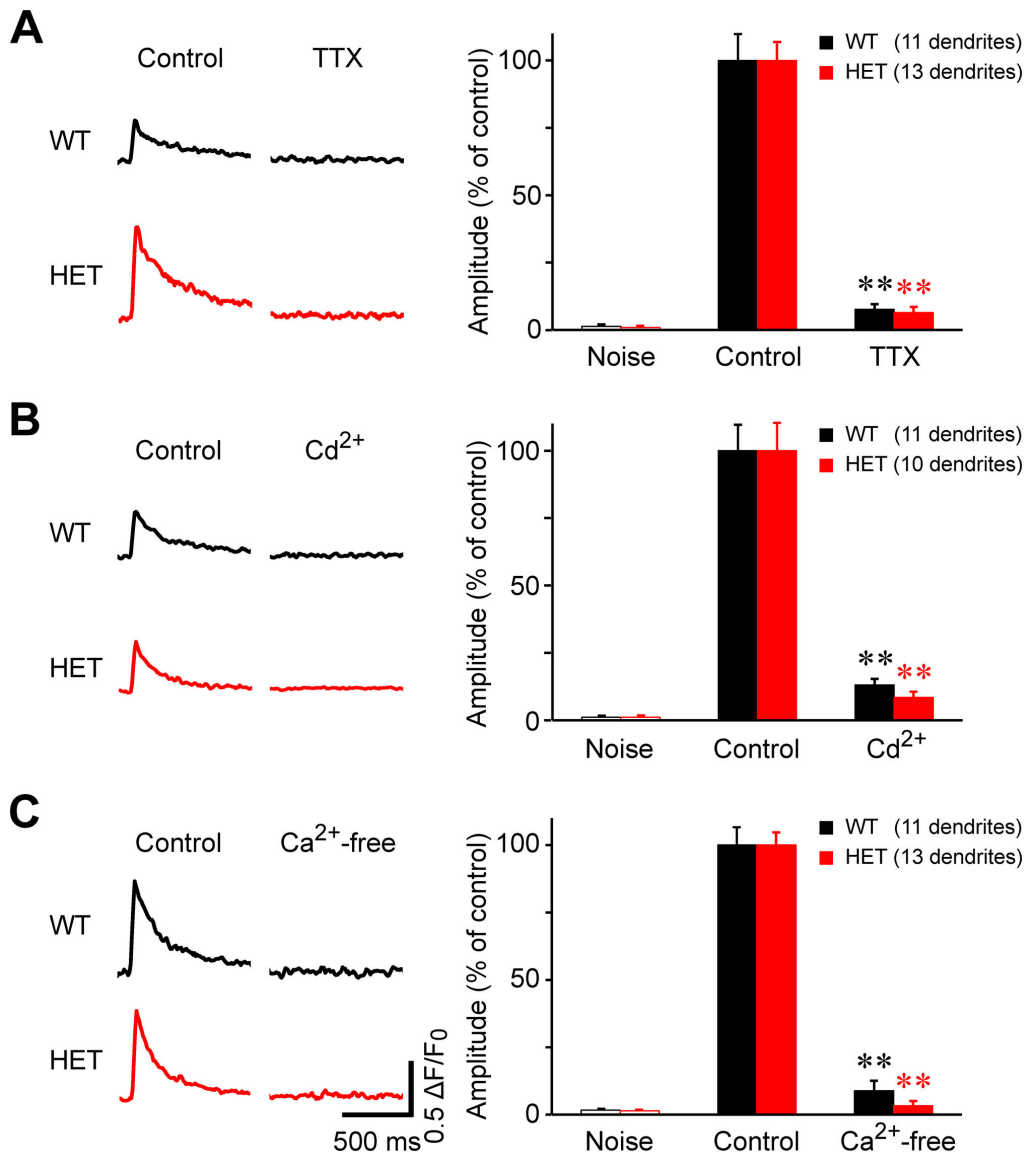
Stimulus-induced Ca^2+^ transients in both wild-type and heterozygous neurons are mediated by the opening of voltage-dependent Na^+^ and Ca^2+^ channels, followed by the influx of extracellular Ca^2+^. (A-C, left) Representative Ca^2+^ transients induced by field stimulation before (control) and during treatment with tetrodotoxin (TTX, 1 μM; A), cadmium ion (Cd^2+^, 200 μM; B), and Ca^2+^-free extracellular solution (C). The scale bar for the traces in panel C applies to panels A-C. (A-C, right) The amplitudes are expressed as the normalized values with respect to the peak amplitude of the control in each neuron. **, p<10^-7^ versus control.

Second, we tested whether the heterozygous knock-in neurons differed from wild-type neurons with respect to the basic mechanisms underlying the evoked [Ca^2+^]_c_ transients in the dendrites (Figure 2). The evoked [Ca^2+^]_c_ transients were suppressed to a level indistinguishable from the pre-stimulus level by the application of tetrodotoxin (TTX), a blocker of voltage-dependent Na^+^ channels (Figure 2A). This result indicates that under control conditions, the electrical stimulus was sufficient to depolarize the neurons and generate a Na^+^-dependent action potential. The [Ca^2+^]_c_ transient was also blocked completely by 200 μM cadmium (Cd^2+^), a blocker of voltage-dependent Ca^2+^ channels (Figure 2B). At this concentration, Cd^2+^ blocks all classes of voltage-dependent Ca^2+^ channels [20,21]. In addition, unlike other divalent metal cations such as Cu^2+^, Cd^2+^ does not quench the fluorescence of Ca^2+^ dyes ([22]; also see Section 19.7 in [23]); therefore, the suppression of the fluorescence signal in the presence of extracellular Cd^2+^ (Figure 2B) is due to blockade of voltage-dependent Ca^2+^ channels. Finally, similar to treating with TTX and blocking the channels with Cd^2+^, replacing the bath solution with Ca^2+^-free solution eliminated the stimulus-induced [Ca^2+^]_c_ rise (Figure 2C), confirming that the rise in control solution is due to Ca^2+^ influx through voltage-dependent Ca^2+^ channels in the cell membrane. 

These three treatments produced a similar effect in wild-type and heterozygous ΔE-torsinA knock-in neurons (p < 10^-7^ for each case of treatment, n=11–13 dendrites for each genotype; Figure 2). These results suggest that the following sequence of events produce the stimulus-induced [Ca^2+^]_c_ transient in both wild-type and heterozygous neurons: i) brief electrical stimulation → *ii*) membrane depolarization → *iii*) opening of voltage-dependent Na^+^ channels → *iv*) generation of an action potential → *v*) opening of voltage-dependent Ca^2+^ channels → *vi*) Ca^2+^ influx from the extracellular solution into the cytoplasm → *vii*) increase in [Ca^2+^]_c_. This sequence is consistent with previous studies of wild-type mice showing that membrane depolarization triggers Ca^2+^ influx into the dendrites of striatal medium spiny neurons via voltage-dependent Ca^2+^ channels [24,25].

### Spatial distribution of [Ca^2+^]_c_ transients

**Figure 3:**
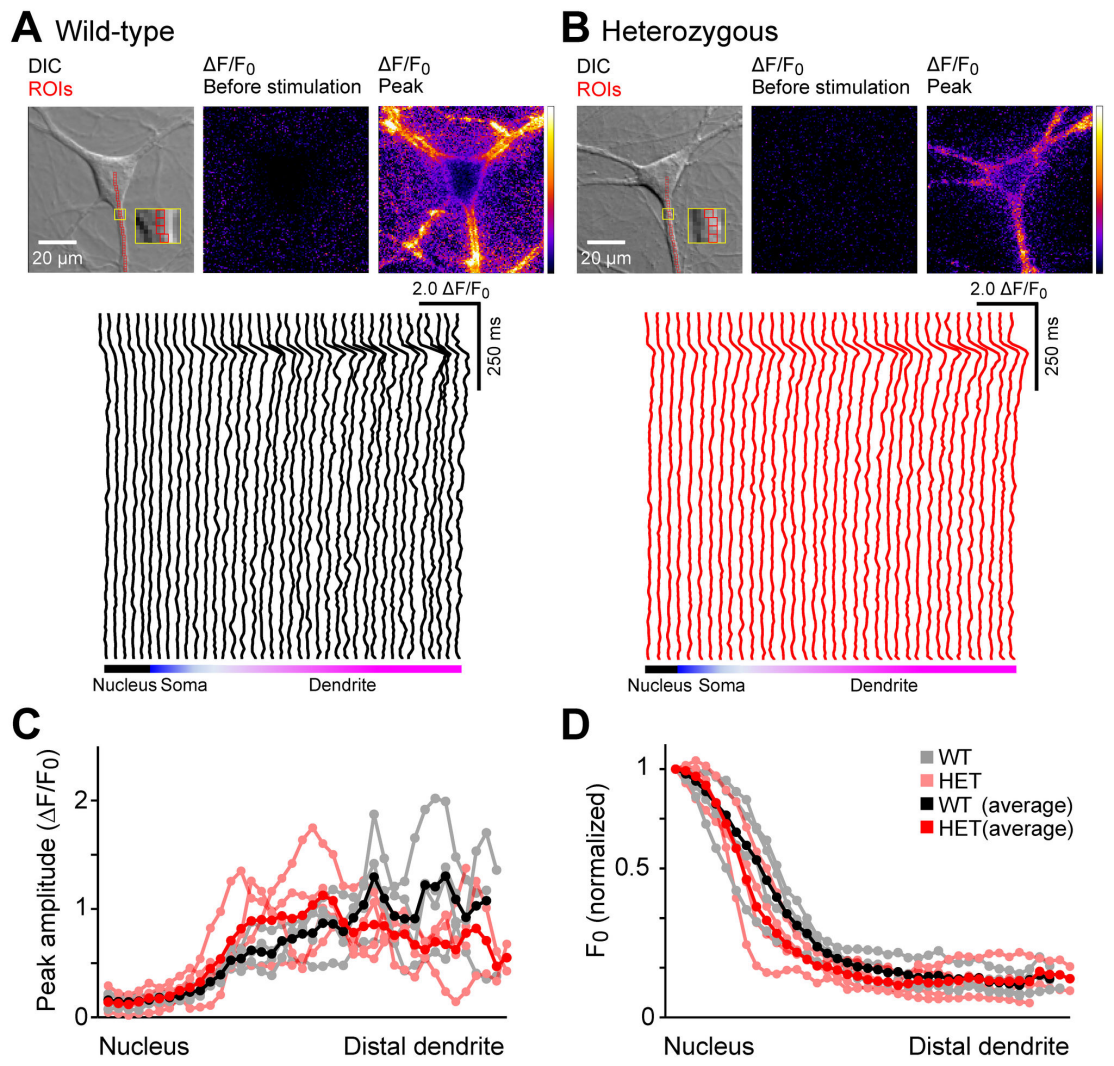
Subcellular [Ca^2+^]_c_ gradients are not affected by the ΔE-torsinA mutation. (A and B) A wild-type (A) and heterozygous neuron (B) analyzed as in Figure 1A, but without spatial averaging. The DIC images show an overlay of a series of individual pixels assigned from the center of the soma (corresponding to the nucleus), through the soma and ending in a dendrite. The insets show a magnified view of the yellow rectangle depicted in the proximal dendrite. The lower panels show the [Ca^2+^]_c_ transients at individual pixels when the neurons were stimulated with a single field-stimulation pulse. The position of the pixels is indicated schematically at the bottom of each trace. (C) The peak amplitudes in individual neurons were plotted against the position relative to the nucleus. (D) Baseline fluorescence intensity (F_0_) in individual neurons was normalized and plotted against the position relative to the nucleus. In panels C and D, each data point represents a pixel. Gray and black symbols represent the raw and averaged data from wild-type neurons, whereas pink and red symbols represent the raw and averaged data from heterozygous neurons.

For the third property of [Ca^2+^]_c_ transients, we evaluated whether the heterozygous neurons differed from wild-type neurons with respect to the spatial distribution of their [Ca^2+^]_c_ transients (Figure 3). The previous analyses (Figures 1 and 2) were based on the fluorescence signal averaged over 12 contiguous pixels in the proximal dendrite. Although this method improved the signal-to-noise ratio through spatial averaging, it could have masked any potential spatial heterogeneity if the ΔE-torsinA mutation altered the subcellular distribution in dendrites. To address this possibility, we examined the [Ca^2+^]_c_ transients in individual pixels without spatial averaging. Small Ca^2+^ events should be detectable because the size of an individual pixel (1.18 μm x 1.18 μm) is comparable to the sizes of elementary Ca^2+^ release events (several microns in diameter) [26] or single spines (~1 μm) [27].

The DIC image was used to define a 1-pixel-wide curve from the nucleus, through the soma and into the dendrite (Figure 3A and B, top left panels; the insets show high-magnification views of the yellow rectangles in the dendrites). Changes in ΔF/F_0_ are shown in image format before stimulation (top center panels) and during the peak of the response (top right panels), and in graphical format for each pixel (Figure 3A and B, bottom panels). Under our experimental conditions, a single field stimulus had little effect on ΔF/F_0_ in the nucleus and somatic cytoplasm. In contrast, the stimulation induced a large increase in the proximal dendrites near the soma, and this increase was progressively larger in more distal regions of the dendrite. Although the ΔF/F_0_ traces were relatively noisy when measured at the individual pixel level, we could not identify any local discrete peaks during the decay phase that would contribute to spatial differences between the heterozygous and wild-type neurons, as these features were common to both wild-type (Figure 3A) and heterozygous (Figure 3B) neurons. To quantify these changes in ΔF/F_0_, peak amplitude was measured and plotted against the pixel number along the curve (Figure 3C). In both heterozygous and wild-type neurons, the more distal dendritic locations had the largest amplitude, likely reflecting an increase in the surface-to-volume ratio that affects the number of Ca^2+^ per unit volume in distal locations. Because F_0_ reflects both the resting [Ca^2+^]_c_ level and the dye concentration, which could affect the measured peak amplitude (i.e., ΔF/F_0_), the F_0_ value was normalized and then plotted against the pixel number along the curve (Figure 3D). This analysis revealed that in both the heterozygous and wild-type neurons, F_0_ was high near the soma and decreased progressively at more distal locations. 

In the analysis shown in Figures 1 and 2, peak amplitude was analyzed by placing ROIs at a predetermined, fixed distance from the soma (7 μm from soma-dendrite border), and a difference in dendritic width at this distance could have affected the results of our analysis. However, we found no statistically significant difference between wild-type (3.22±0.10 μm, n=84 dendrites) and heterozygous dendrites (3.23±0.09 μm, n=84 dendrites; p>0.9). Moreover, at this same location, we found no difference with respect to F_0_ values between the wild-type (299.4±21.4 arbitrary units (a.u.), n=84) and heterozygous neurons (388.8±39.9 a.u., n=84; p>0.05).

The single-pixel analysis revealed that the subcellular spatial differences in [Ca^2+^]_c_ were similar between the wild-type and heterozygous neurons. Specifically, both genotypes i) lacked local discrete peaks during the decay phase, ii) had the largest peak amplitude at the more distal observation points, and iii) a similar tendency for smaller F_0_ values at the more distal observation points.

### [Ca^2+^]_c_ transients in response to a train of stimuli

**Figure 4:**
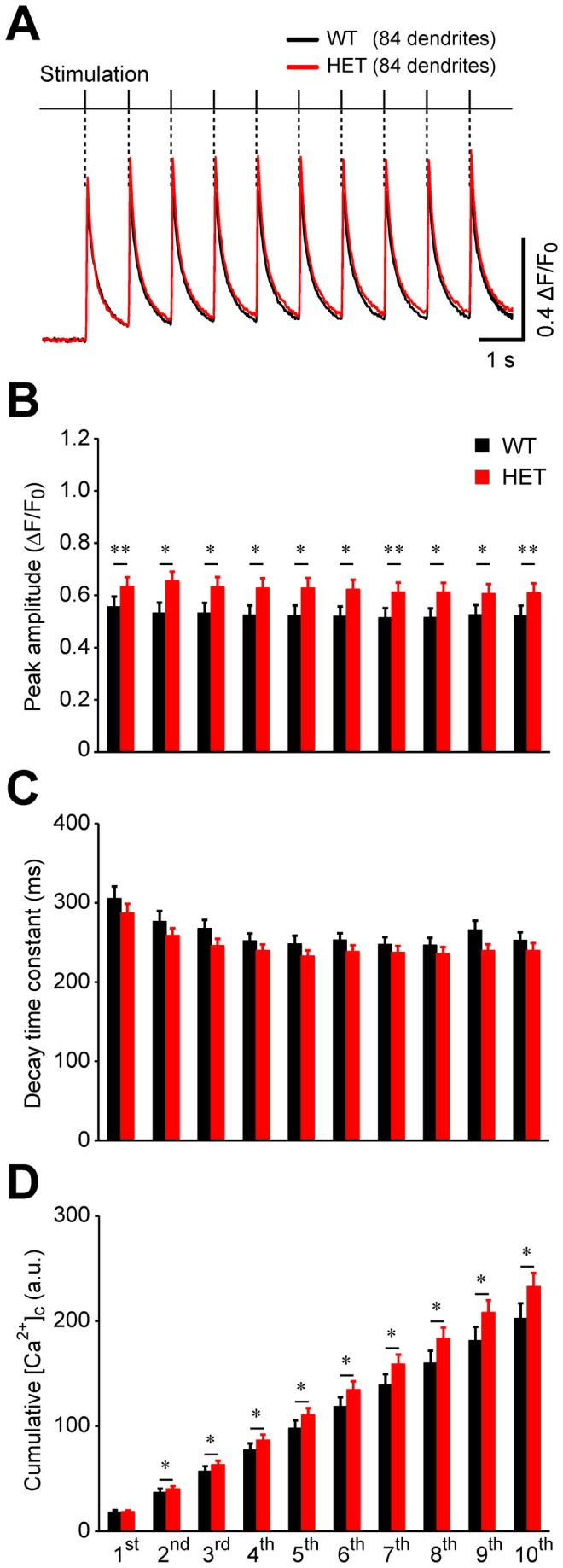
There was a difference in stimulus-train-induced [Ca^2+^]_c_ dynamics between wild-type and heterozygous neurons. (A) Average traces of [Ca^2+^]_c_ transients in response to 10 stimuli applied at 1 Hz. The wild-type trace is shown in black, and the heterozygous trace is shown in red. Error bars are too small to be visible. (B) Summary of the peak amplitudes in response to each pulse in the 10-pulse train. *, p<0.05; **, p<0.01. (C) Summary of the decay time constants after each peak in response to the indicated pulse number. In either genotype, the time constants of the 3^rd^ through the 10^th^ pulse were significantly different from the time constant of the 1^st^ pulse (p<0.05). However, there was no genotypic difference. (D) Cumulative change in [Ca^2+^]_c_ in response to the indicated pulse number. Significant difference was detected between the wild-type and heterozygous neurons at pulses 2-10 (*, p<0.05; n=84 dendrites for both wild-type and heterozygous neurons).

Our fourth set of experiments was designed to evaluate how heterozygous and wild-type neurons handle increased [Ca^2+^]_c_ loads, by stimulating neurons with a train of stimuli. Similar to our results obtained with a single pulse (see Figure 1B), stimulating the neurons with 10 pulses at 1 Hz induced [Ca^2+^]_c_ transients that were synchronized with each stimulus in the train (Figure 4A). 

The peak amplitudes in the 10-pulse protocol showed a statistically significant difference between the heterozygous and wild-type neurons for all the pulses (p<0.03 for each of the ten pulses) (Figure 4B). For both the heterozygous and wild-type neurons, the decay time constants of the 3^rd^ through the 10^th^ pulses were significantly faster than the first time constant (p<0.05), reflecting an accelerated decay with multiple pulses (Figure 4C). However, there was no significant difference in the time constant between heterozygous and wild-type neurons at any pulse (p>0.1 for each of the ten pulses). As a lumped parameter, including both the amplitude and decay time course, we measured the cumulative change in [Ca^2+^]_c_. This was achieved by measuring the absolute area under the [Ca^2+^]_c_ transients starting from the time when the stimulus was applied until 1 sec after each pulse (Figure 4D). The analysis revealed a statistically significant difference between heterozygous and wild-type neurons for the 2^nd^ through 10^th^ pulses (p<0.05 for each of the nine pulses). At the end of the 10^th^ pulse, the mean values for the heterozygous and wild-type neurons were 233 and 203 a.u., respectively with ~15% increase. Taken together, these results suggest a slight genotypic difference in [Ca^2+^]_c_ transients evoked by a 1-Hz train of stimuli.

### Fractions of neurons that responded with a [Ca^2+^]_c_ transient

**Figure 5:**
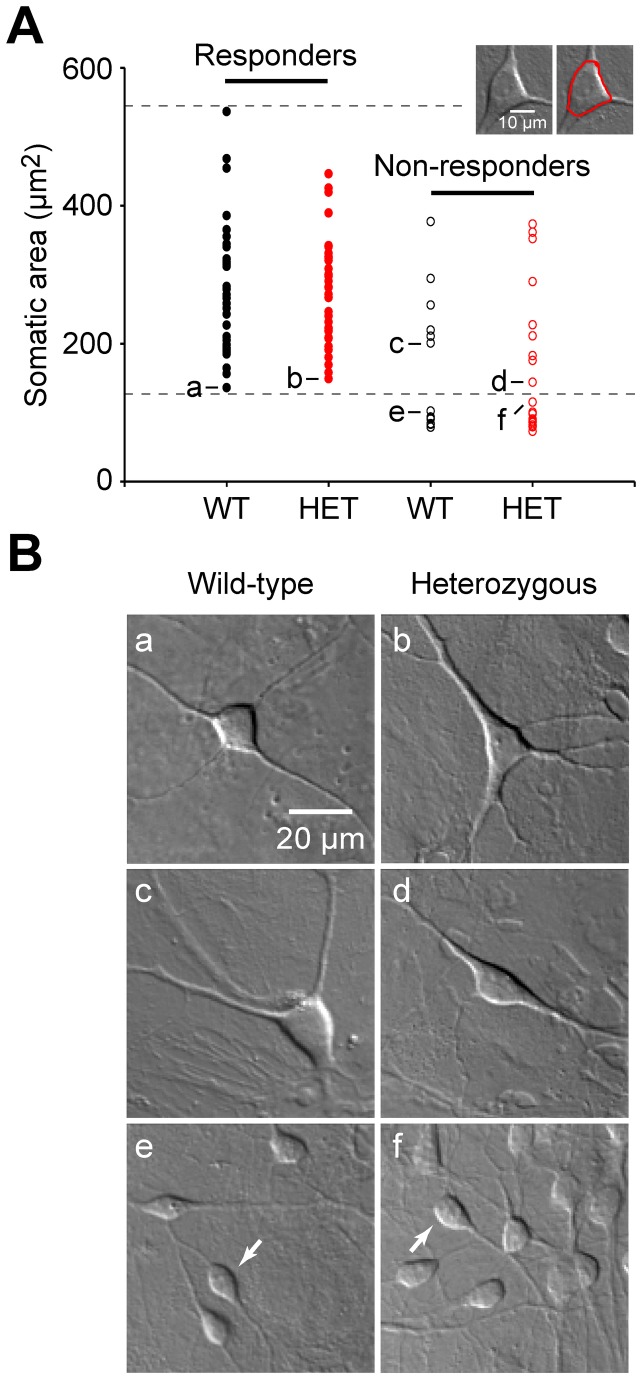
Wild-type and heterozygous neurons have a similar percentage of neurons that responded with stimulus-induced [Ca^2+^]_c_ transients. (A) Somatic area is plotted for individual wild-type and heterozygous neurons that responded (responders, left) or did not respond (non-responders, right) to the 10-pulse stimuli. The horizontal dashed lines show the range of somatic areas in which responding neurons were detected in wild-type neurons (158-537 μm^2^). (B) Images of the neurons indicated in panel A by the corresponding alphabetical letters. Note that the images in panels a through f were captured at the same magnification; panels e and f contain clusters of small cells (arrows).

Because some neurons failed to respond to field stimulation under our experimental conditions, we next examined the proportion of neurons in which we could observe a [Ca^2+^]_c_ transient as the fifth property (Figure 5). The 10-pulse protocol elicited detectable [Ca^2+^]_c_ transients in wild-type neurons of various sizes (Figure 5A; each filled black circle represents a responding neuron). By measuring somatic area with an ROI around the soma (Figure 5A, inset), we found that the responding wild-type neurons ranged in size from 158.03 to 536.68 μm^2^ (horizontal dotted lines), with an average size of 269.70±15.23 μm^2^ (n=37 neurons). The responding heterozygous neurons covered a similar range (filled red circles) with an average size of 271.23±12.60 μm^2^ (n=37 neurons) (p>0.9 vs. wild-type). The non-responding neurons also varied in size (Figure 5A, open circles; see also Figures S1 and S2 in File S1), but all neurons smaller than 158 μm^2^ did not respond; the lack of detectable responses in these relatively small neurons could be attributed to a small signal-to-noise ratio in some neurons. When the small neurons (i.e., those below the lower dotted line) were excluded from the analysis, the percentage of wild-type responders (86%, 37 out of 43 neurons) was similar to the percentage of heterozygous responders (80%, 37 out of 46 neurons). Moreover, we found no gross morphological difference between the responding and non-responding neurons for either genotype (Figure 5B). 

### Correlations among the [Ca^2+^]_c_ transient parameters

**Figure 6:**
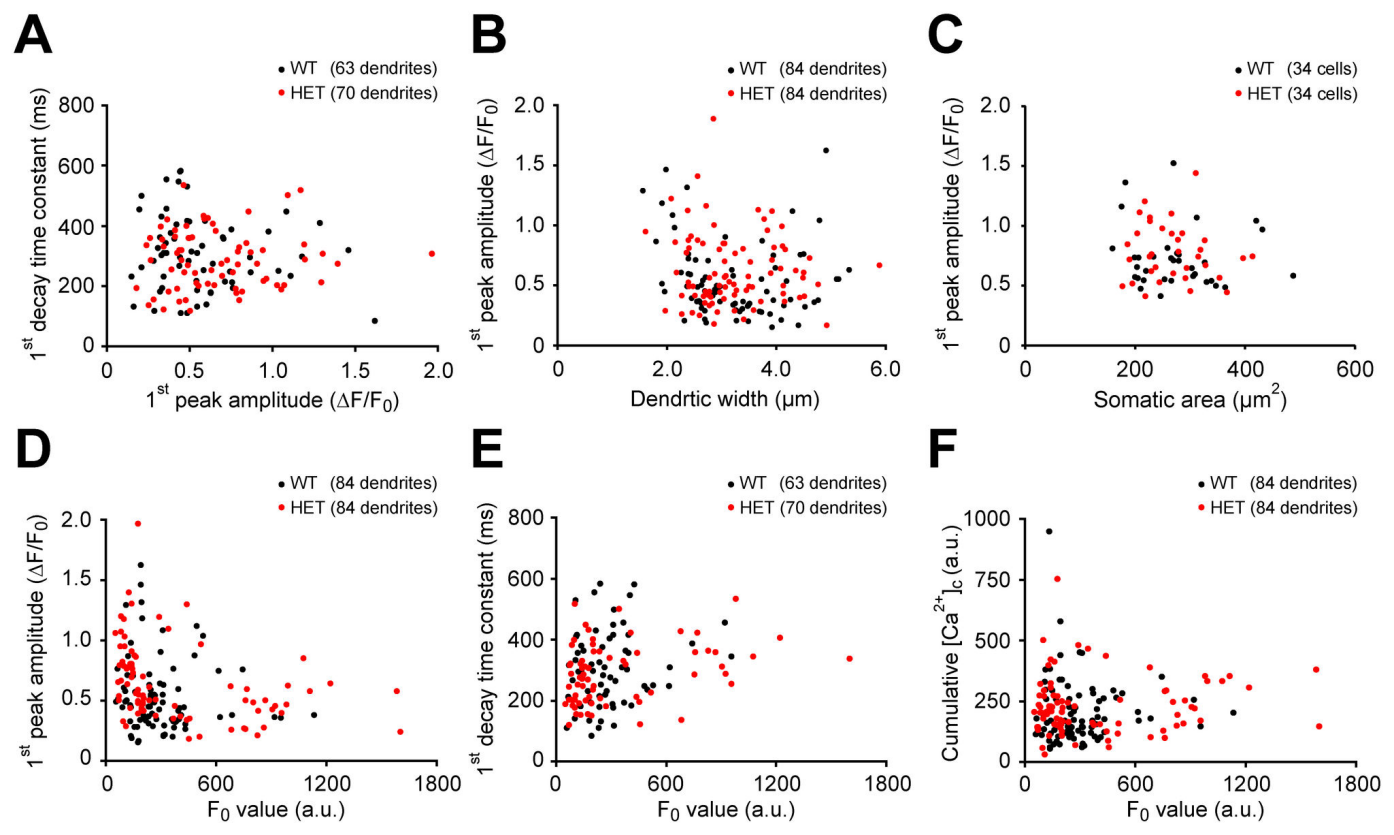
Neither the wild-type nor heterozygous neurons showed a correlation among the various [Ca^2+^]_c_ parameters. (A) The decay time constant is plotted against the peak amplitude of the first response (correlation coefficient r=0.124, p=0.333 for wild-type and r=0.047, p=0.700 for heterozygous neurons). (B) The amplitude of the first peak is plotted against dendrite width (r=0.121, p=0.274 for wild-type and r=0.079, p=0.477 for heterozygous neurons). (C) The amplitude of the first peak is plotted against somatic area (r=0.080, p=0.653 for wild-type and r=0.104, p=0.559 for heterozygous neurons). (D) The amplitude of the first peak amplitude is plotted against F_0_ (r=0.140, p=0.203 for wild-type and r=0.198, p=0.071 for heterozygous neurons). (E) The decay time constant of the first pulse is plotted against F_0_ (r=0.169, p=0.186 for wild-type and r=0.230, p=0.055 for heterozygous neurons). (F) The cumulative [Ca^2+^]_c_ change at the end of 10^th^ response is plotted against F_0_ (r=0.010, p=0.928 for wild-type and r=0.071, p=0.521 for heterozygous neurons).

Finally, to further test whether the heterozygous neurons differ from wild-type neurons with respect to their [Ca^2+^]_c_ transients, we analyzed the correlation between the parameters of the transients, as a subtle change could have been obscured by the population analysis of each parameter. Despite this detailed analysis, we found no statistically significant difference in the correlations for either genotype. Specifically, no correlation was found between peak amplitude and the decay time constant in response to the first stimulus (p>0.05, Figure 6A), between dendritic width and the first peak amplitude (p>0.05, Figure 6B), or between somatic area and the first peak amplitude (p>0.05, Figure 6C). In addition, we found no correlation between somatic area and dendritic width (p>0.05, Figure S3 in File S1). Lastly, the F_0_ values were not significantly correlated with first peak amplitude (Figure 6D), the first decay time constant (Figure 6E), or the cumulative [Ca^2+^]_c_ change after the 10^th^ pulse (Figure 6F).

### GABAergic and cholinergic neurons in striatal cultures

**Figure 7:**
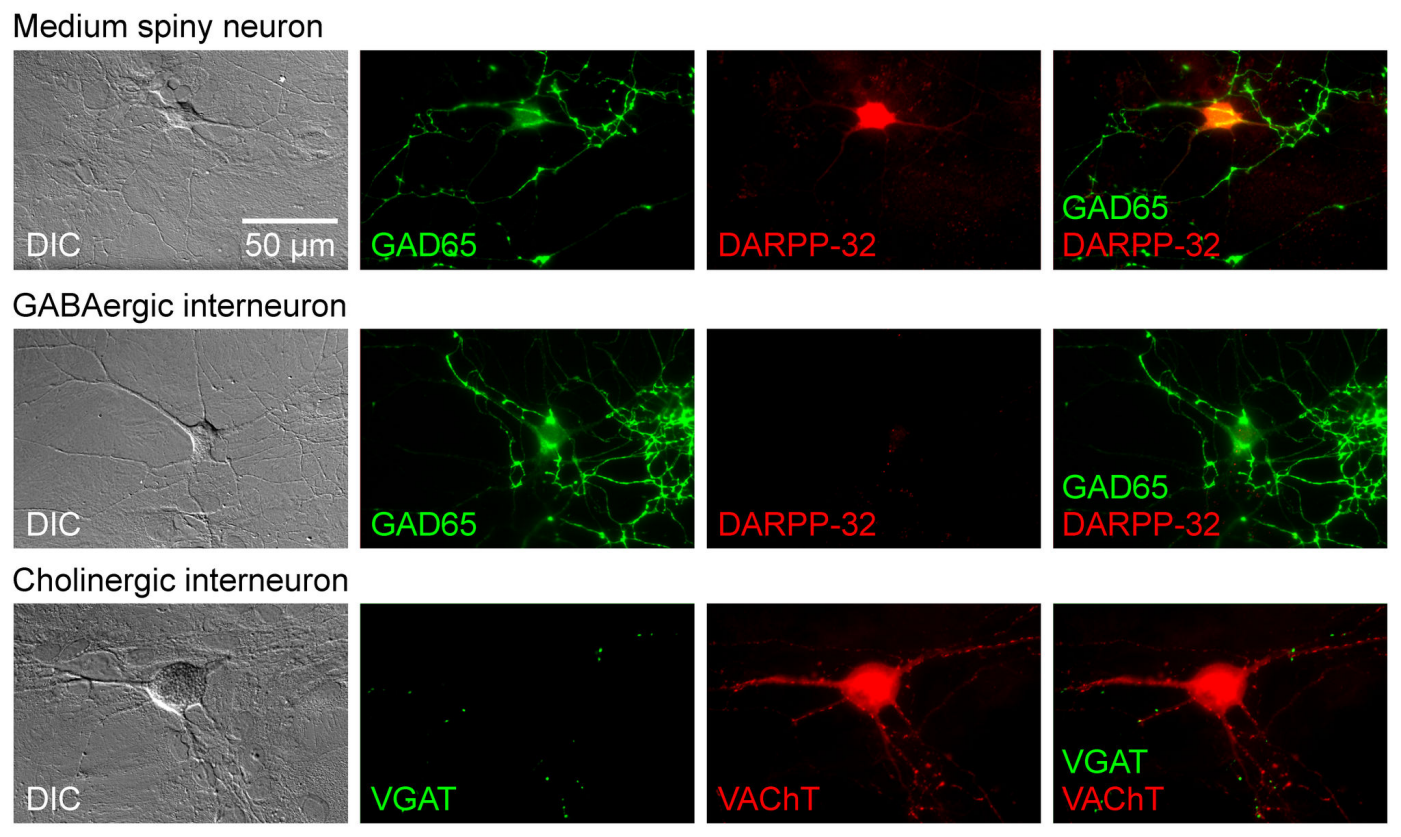
Cultured striatal cells were identified by their immunocytochemistry profile. Markers of GABAergic cells (GAD65), medium spiny neurons (DARPP-32), and cholinergic cells (VAChT) were used to classify the cells as medium spiny neurons (GAD65-positive/DARPP-32-positive), GABAergic interneurons (GAD65-positive/DARPP-32-negative) or cholinergic interneurons (GAD65-negative or VGAT-negative/VAChT-positive). In each row, a cell was imaged with DIC optics (left), two fluorescence channels, and was shown in a merged fluorescence image (right). The scale bar in the top-left panel applies to all panels. These images were obtained from three heterozygous neurons; similar results were obtained from wild-type neurons (data not shown).

Next, we used immunocytochemistry to identify the types of striatal neurons for which we had characterized the Ca^2+^ dynamics (Figure 7). Our analysis identified three types of cells that were present in the striatal cultures. In the first type, the cell body was positive for both the GABAergic marker, glutamic acid decarboxylase 65 kDa (GAD65) [28] and the medium spiny neuron marker, dopamine- and cAMP-regulated phosphoprotein 32 kDa (DARPP-32) [29] (Figure 7, top row). In the second type, the cell body was positive for GAD65 but negative for DARPP-32 (Figure 7, middle row). In the third cell type, the cell body was negative for the GABAergic markers GAD65 or vesicular GABA transporter (VGAT) [30], but the cell body was positive for the cholinergic marker, vesicular acetylcholine transporter (VAChT) (Figure 7, bottom row). GAD65 and VAChT strongly labeled the cellular processes and nerve terminals, and therefore relatively diminished the labels in the cell bodies (Figure 7). However, the diffuse staining of cell bodies is significantly intense in comparison to background fluorescence (to be numerically evaluated in the next section). DARPP-32 stained the cell bodies in a diffuse manner. These findings made the cellular identification possible by observing the somatic staining intensity. 

**Figure 8:**
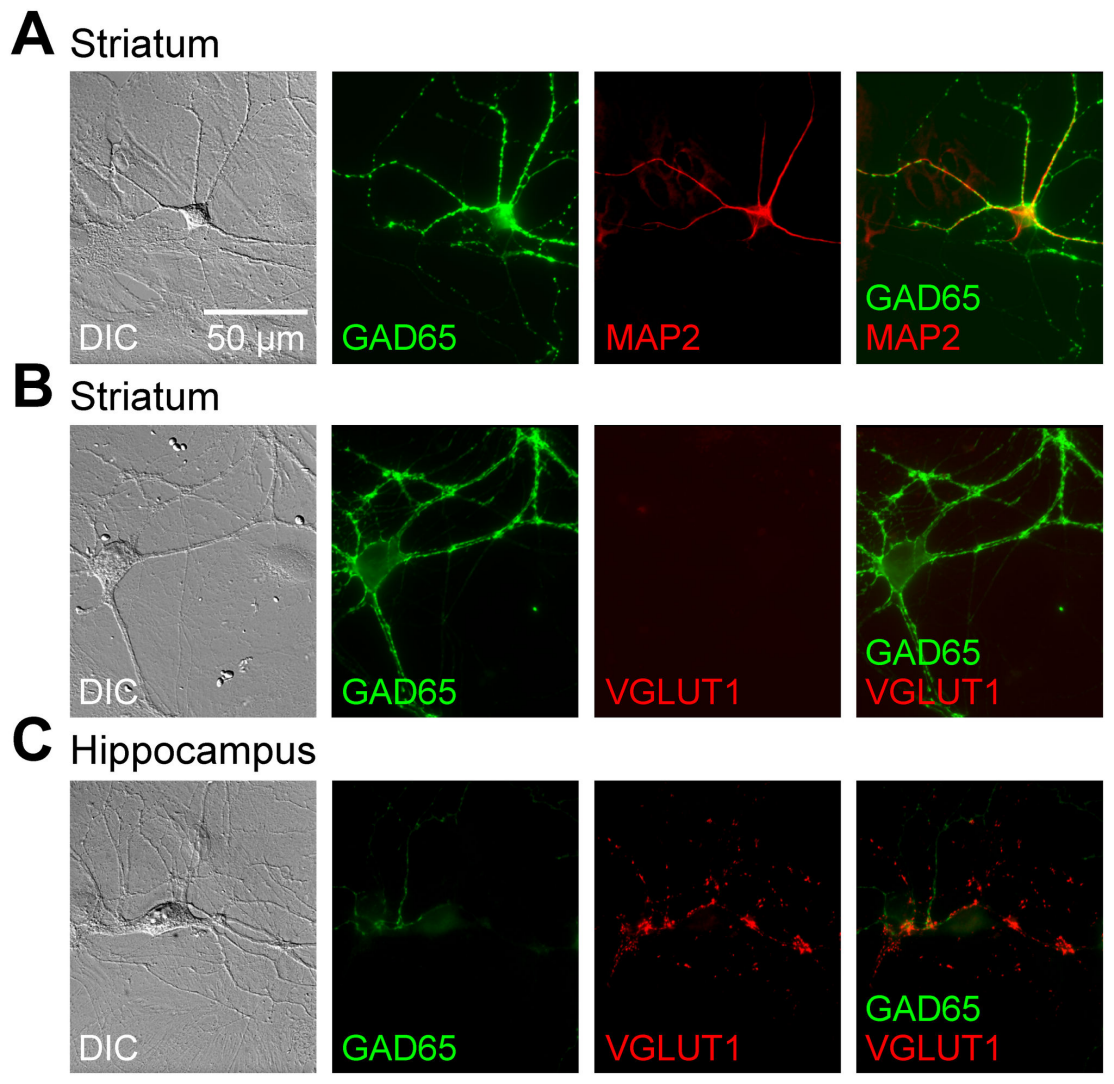
Further characterization of cultured striatal cells. (A) A representative wild-type cell was positive for GAD65 and for MAP2, indicating that this cell is a GABAergic neuron. (B) A representative field in the wild-type striatal culture contained GAD65-positive structures such as nerve terminals and soma, but lacked VGLUT1-positive glutamatergic structures. (C) In comparison, wild-type hippocampal neurons in culture contain many nerve terminals that are positive for VGLUT1 and GAD65. In each row, a cell was imaged with DIC optics (left), two fluorescence channels, and was shown in a merged fluorescence image (right). The scale bar in the top-left panel applies to all panels. Similar results were obtained from heterozygous neurons (data not shown).

These cells were confirmed to be neurons by their positive staining with the dendritic marker, microtubule-associated protein 2 (MAP2) (Figure 8A for GABAergic neurons; data not shown for the cholinergic neurons). In our striatal cultures, no structures were positive for the glutamatergic marker, vesicular glutamate transporter 1 (VGLUT1) (Figure 8B). This finding is in contrast to cultured hippocampal neurons, in which the nerve terminals [31] and somata of glutamatergic neurons were positive for VGLUT1 (Figure 8C). Similar results were obtained from cultures of wild-type and heterozygous ΔE-torsinA knock-in neurons (Figures 7 and 8, and data not shown). These results indicate that our dissociated striatal cultures are composed of GABAergic medium spiny neurons, GABAergic interneurons, and cholinergic interneurons, qualitatively similar to the distribution of striatal neurons *in situ* [2,3,32]. 

### Proportions of striatal GABAergic neurons, including medium spiny neurons and GABAergic interneurons

**Figure 9:**
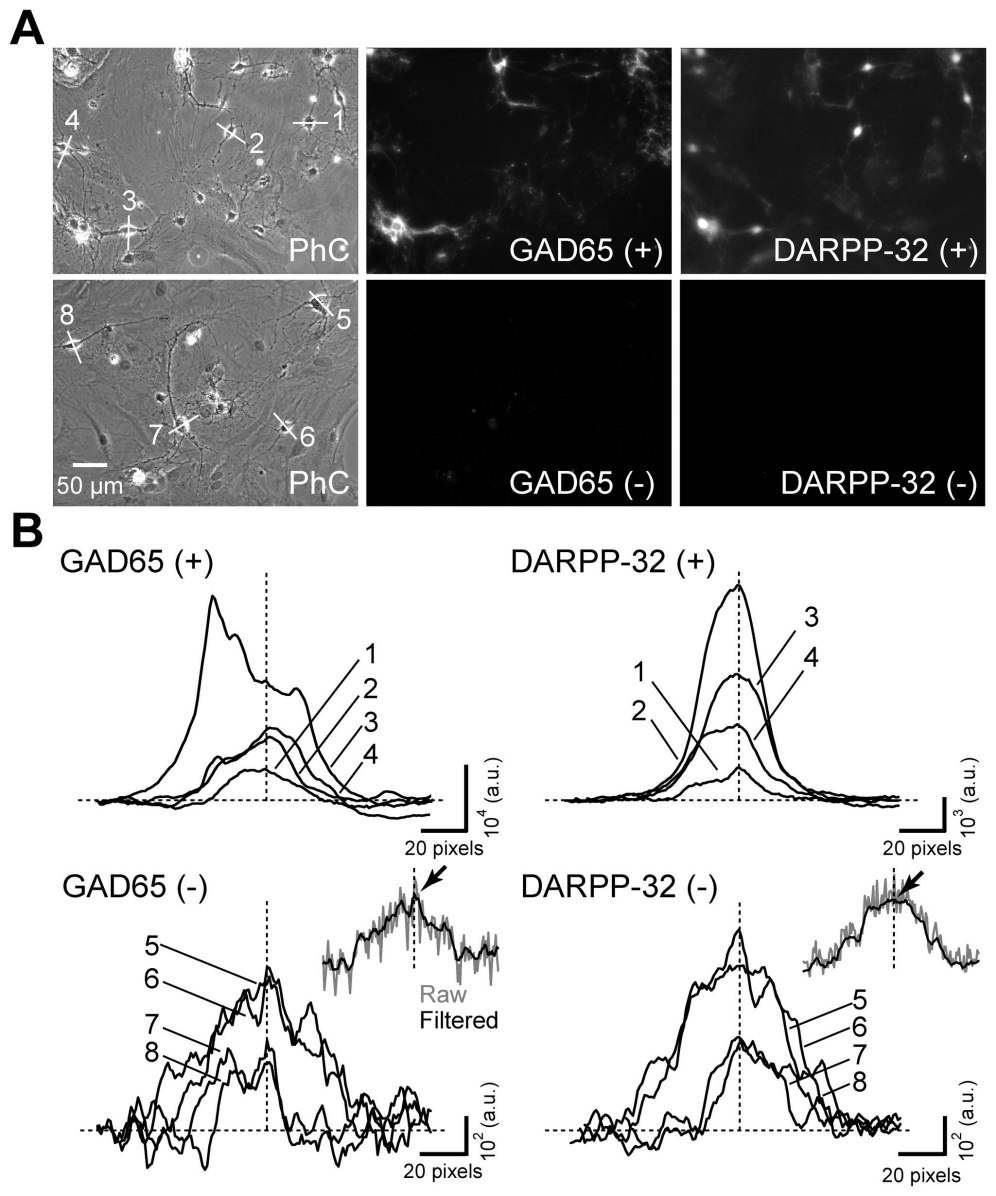
Variable fluorescence intensities of neurons. (A, top) Cultured striatal neurons were stained with the anti-GAD65 and anti-DARPP-32 antibodies. (A, bottom) As a negative control, cultured neurons were stained in the absence of the primary antibodies and were imaged under the same conditions as in top row. (+) and (-) represent the presence and absence of primary antibodies in this figure. In each row, a field was imaged with the phase-contrast optics (PhC, left), and two fluorescence channels. The scale bar in the bottom-left panel applies to all panels. (B) Fluorescence intensity along the lines indicated in the fluorescence images in panel A. Horizontal dotted lines indicate the fluorescence intensity levels in the surrounding background areas. Vertical dotted lines indicate the aligned positions of the somatic centers, where the fluorescence intensity was measured. Note that the perimeter of the GAD65 (+) trace (#3, brightest signal) shows a signal more intense than the somatic center, due to the presence of GABAergic nerve terminals on the surface of the soma. Also note that all traces were running-averaged to remove noise, as indicated in the bottom insets for negative controls. Gray and black traces represent raw and average data, with the arrow pointing at the measured somatic intensity level.

To objectively measure the relative ratios of medium spiny neurons and GABAergic interneurons, the cultures were double labeled for GAD65 and DARPP-32, and the staining intensities were measured in the soma. Each neuron was assessed for positive or negative labeling of each antigen based on whether the measured intensity was above that antigen’s threshold value (see Materials and Methods). Fluorescence intensity was measured as an average intensity of the soma measured along a line (Figure 9A). This line-intensity analysis is an effective method for obtaining information regarding the stained structures [33]. At a qualitative level, the staining intensity of GAD65 and DARPP-32 varied among the neurons (Figure 9B). Some neurons were clearly visible (Figures 7 and 9) and were stained with sufficient intensity to be readily identified as being positive for both GAD65 and DARPP-32 (e.g. cell 3 in Figure 9A, B). On the other hand, the staining of some neurons was similar to the background intensity measured from a negative control in which the primary antibody was omitted. The staining intensity of some cells was dependent on the size of the cell, especially in negative control (Figure 9B).

**Figure 10:**
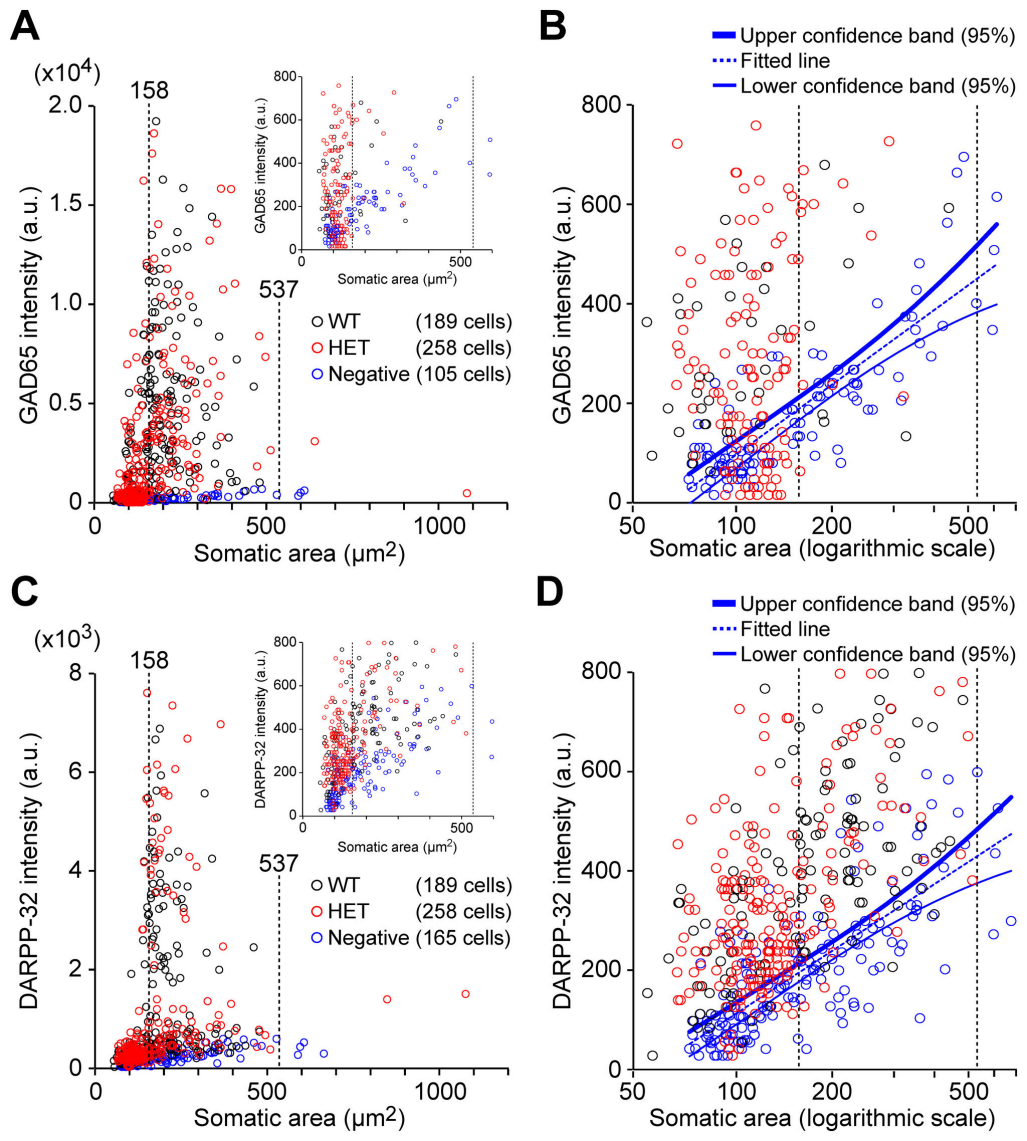
Fluorescence intensity in the soma was used to identify neurons that stained positive for the indicated marker. (A) Plot of fluorescence intensity in the GAD65 channel against somatic area; each point indicates an individual neuron. Inset shows an expanded view. The black and red circles represent wild-type and heterozygous neurons, respectively, stained with the primary antibody. The blue circles represent negative controls pooled from wild-type and heterozygous neurons, and stained without the primary antibody. The vertical dashed lines indicate the range of somatic area (158-537 μm^2^) that corresponds to neurons that responded to field stimulation (see Figure 5A). (B) The same plot as the inset from panel A, with the *x*-axis plotted on a logarithmic scale to the base 10. The solid blue curves indicate the upper (thick) and lower (thin) limits of the 95% uniform confidence band of negative control intensity. The dashed blue line indicates the regression line. The upper limit was used as an intensity threshold for determining whether a neuron was positive or negative for GAD65. (C and D) A similar analysis as panels A and B, for DARPP-32 staining.

To analyze the data quantitatively, staining intensity was plotted against somatic area (Figure 10). The vertical dashed lines indicate the somatic area range that we measured for Ca^2+^ responders (158-537 μm^2^, see Figure 5A). The intensity of GAD65 (Figure 10A) and DARPP-32 (Figure 10C) staining in many of the wild-type (black circles) and heterozygous (red circles) neurons was stronger than the intensity of neurons stained in the negative control conditions (blue circles). The insets show the data on an expanded y-axis (from 0-800 a.u.), showing that the distinction between positive and negative cells was not clear in this range. The intensity threshold was then obtained from the intensity of the cellular background signal in the negative controls. Because the background signal differed by somatic area (Figure 9B), rather than using a fixed threshold, the intensity threshold was defined as the upper limit of the 95% uniform confidence band of the negative controls (thick blue curves in Figure 10B and D).

The immunocytochemistry results are summarized in Table 1 for neurons with somata in the range of 158-537 μm^2^. GAD65-positive (i.e., GABAergic) neurons comprised 97.5% (117/120 neurons) of the wild-type striatal neurons and 97.9% (94/96 neurons) of the heterozygous striatal neurons. In addition, DARPP-32-positive neurons comprised 95.0% (114/120 neurons) of the wild-type striatal neurons and 92.7% (89/96 neurons) of the heterozygous striatal neurons. Thus, the vast majority of cultured striatal neurons are GABAergic medium spiny neurons (i.e., positive for both GAD65 and DARPP-32), and the minority of cells are GABAergic interneurons (i.e., positive for GAD65 but negative for DARPP-32). These percentages are similar to the reported values for striatal GABAergic neurons *in vivo* [2,3,32] and *in vitro* [28]. However, a previous study of cultured striatal neurons reported a lower percentage of DARPP-32-positive neurons [34]. This difference may be due to the quantitative analysis used in our study and/or differences in culture conditions.

**Table 1:** Distribution of the various populations of cultured striatal neurons.

	Medium spiny neuron	GABAergic interneuron	Cholinergic interneuron
Wild-type	**95.0%** (114/120)	**2.5%** (3/120)	**1.0%** (1/104)
Heterozygous	**92.7%** (89/96)	**5.2%** (5/96)	**1.4%** (3/217)

The numbers in parentheses indicate the numbers of neurons.

### Cholinergic interneurons in striatal culture

Our striatal cultures contained few cholinergic interneurons. The mean somatic area of the VAChT-positive cholinergic interneurons was 653 μm^2^ in wild-type mouse (n=1) and 773.9 ± 22.6 μm^2^ in heterozygous mouse (n=3) with a range of 746-819 μm^2^, exceeding those of GABAergic neurons that were used for Ca^2+^ imaging experiments. Neurons were clearly distinguished into two discrete populations of cells that were either positive or negative for the cholinergic marker. The percentages of cholinergic neurons in the wild-type and heterozygous cultures were 1.0 and 1.4% (Table 1), and these percentages are similar to the 0.5-2% for striatal neurons *in situ* [35]. These results indicate that the Ca^2+^ dynamics that we analyzed were most likely measured primarily in medium spiny neurons and GABAergic interneurons, with only a minor—and likely negligible—contribution of cholinergic interneurons.

## Discussion

Most of the striatal neurons that were cultured from the wild-type and heterozygous ΔE-torsinA knock-in mice were GABAergic, with 93-95% medium spiny neurons and 3-5% GABAergic interneurons. When these GABAergic neurons were stimulated using electrical field stimulation, heterozygous neurons showed an increase in the peak amplitude of the dendritic [Ca^2+^]_c_ transients and consequently the cumulative change in [Ca^2+^]_c_. There were no changes in the other features, namely, the decay time course of [Ca^2+^]_c_ transients, mechanisms of inducing [Ca^2+^]_c_ transients, subcellular gradients in [Ca^2+^]_c_ transients, properties of the neurons that responded to a stimulus, and correlations between these parameters. Because the peak amplitude of the [Ca^2+^]_c_ transient is determined by the coordinated activity of voltage-dependent Na^+^ and Ca^2+^ channels, the dynamics of the [Ca^2+^]_c_ transient can be used as a pooled readout of neuronal excitability that is independent of synaptic activity. Thus our results indicate a slight increase in the excitability of heterozygous neurons. In addition, because the decay phase of the [Ca^2+^]_c_ transient reflects the kinetics of the mechanisms that remove Ca^2+^ from the cytoplasm to the extracellular space (clearance) and/or to intracellular Ca^2+^ stores (sequestration) [36], the lack of overt difference in [Ca^2+^]_c_ decay time course between wild-type and heterozygous neurons indicates that there is no overt abnormality in the Ca^2+^-removal properties in striatal GABAergic neurons in ΔE-torsinA knock-in mice. 

### Comparison with previous studies

A recent report found that the electrical excitability of striatal cholinergic interneurons in heterozygous ΔE-torsinA knock-in mice is similar to wild-type neurons [16]. This report is supported by previous studies that reported a similar lack of electrical excitability change in other models of DYT1 dystonia. Specifically, in transgenic mice overexpressing ΔE-torsinA, no change in excitability was detected in the striatal neurons, including medium spiny neurons [37,38], fast-spiking GABAergic interneurons [38], and cholinergic interneurons [39,40]. The results obtained with striatal medium spiny neurons were replicated recently in transgenic rats overexpressing ΔE-torsinA [41]. Furthermore, in conditional knock-out mice in which torsinA is deleted selectively in cholinergic neurons, no change in electrical excitability was detected in striatal cholinergic interneurons or substantia nigra dopaminergic neurons [42]. Thus, the electrical excitability of striatal neurons is largely unaffected by drastic changes in the torsinA protein expression, either under overexpression or conditional knock-out. If this body of work is a guide, our result on [Ca^2+^]_c_ dynamics indicates an increased activity or expression of Ca^2+^ channels. In support of this notion, the expression of N-type voltage-dependent Ca^2+^ channels is increased in the striatal cholinergic interneurons of transgenic mice overexpressing ΔE-torsinA [39,40].

### Minimal Ca^2+^ overload

Excess Ca^2+^ overload in striatal neurons can lead to neurodegeneration, for example in the case of medium spiny neurons cultured from a Huntington’s disease model [43,44]. Moreover, 3-nitropropionic acid, an irreversible inhibitor of mitochondrial respiratory Complex II (succinate dehydrogenase), induces Ca^2+^ overload in striatal neurons when glutamate receptors are activated [45], which could contribute to striatal degeneration [46] and at least one form of dystonia [47]. Our results showed a statistically significant increase in the Ca^2+^ load during a train of stimuli. However, this load seems to be of minor degree, because previous studies found no detectable neurodegeneration in the brains of DYT1 dystonia patients [48-51] or in the striatum of heterozygous ΔE-torsinA knock-in mice [14,52].

### Changes in synaptic activity and morphological features of striatal neurons

Striatal medium spiny neurons form the principal output pathway of the striatum. Both the electrical activity and the release of synaptic GABA from medium spiny neurons critically determine the synaptogenesis and wiring strength within the multi-stage network of the basal ganglia, as revealed by the effect of network activity on various parameters such as the frequency of spontaneous glutamatergic input and the density of dendritic spines [6]. Our data showing a slight increase in [Ca^2+^]_c_ dynamics of striatal GABAergic neurons, including medium spiny neurons, indicate a possibility that the change can influence the synaptic transmission within striatum.

It is worth noting that such functional changes have been reported in heterozygous ΔE-torsinA knock-in mice. For example, these mice lack long-term depression at their cortico-striatal glutamatergic synapses [15], have a shortened period of suppressed neuronal firing due to thalamo-striatal glutamatergic stimulation [16], and have suppressed dopamine release in striatum [17]. These findings are supported by the accumulating body of data obtained using transgenic mice and rats that overexpress ΔE-torsinA. For example, long-term depression is abolished at cortico-striatal synapses [37,41,53], whereas long-term potentiation is enhanced [37,53] and cannot be reverted to the baseline, non-potentiated level (lack synaptic depotentiation) [37,41,53]. Furthermore, intra-striatal GABAergic transmission is enhanced [38] and dopaminergic transmission is suppressed [54,55]. These functional changes are accompanied by subtle but specific morphological changes in the striatal synapses of the knock-in mice. A recent study revealed that the subcellular locations of glutamatergic and dopaminergic synapses are altered [52], and the medium spiny neurons have fewer dendrites and fewer dendritic spines [52] without a change in spine density [15,52]. These results provide evidence that the mutation perturbs the activity of striatal synaptic transmission. It is plausible that the altered [Ca^2+^]_c_ dynamics are involved in certain aspects of these synaptic changes, although precise mechanisms are not clear at present. 

In contrast to these synaptic alterations, other morphological changes are not robust enough to overtly affect excitability. The size of the striatal GABAergic interneurons differed from wild-type counterparts in the knock-in mice: the somata of “fast-spiking” parvalbumin-expressing interneurons were larger, whereas the somata of “low-threshold spiking” neuronal nitric oxide synthase (nNOS)-expressing GABAergic interneurons were smaller than wild-type [52]. However, the size of the striatal medium spiny neurons in the knock-in mice was unchanged [52]. Consistently, our data revealed no overt differences in soma size, and this is likely because our cultured neurons include a mixed population of GABAergic neurons that contains primarily medium spiny neurons. In addition, we found no correlation between the amplitude of the [Ca^2+^]_c_ transient and either soma size or dendrite width in either heterozygous or wild-type neurons, suggesting that these neurons have no overt morphological changes that affect their [Ca^2+^]_c_ dynamics.

It would be informative to investigate whether restoring the changes in the [Ca^2+^]_c_ dynamics, excitability and synaptic transmission within the striatum can be an effective strategy for treating patients with DYT1 dystonia. Whether the ΔE-torsinA mutation affects the structure and function of basal ganglia network other than the striatum is currently not well known and also merits future investigation. 

## Materials and Methods

### Ethics Statement

Animal care and procedures were approved by the University of Iowa Animal Care and Use Committee (IACUC permit numbers 1110226, 1204080, 1304076) and were performed in accordance with the standards established by the National Institutes of Health Guide for the Care and Use of Laboratory Animals (NIH Publication No. 80-23, revised 1996). Every effort was made to minimize suffering of the animals.

### Genotyping

On postnatal day 0–1, pups of either gender from DYT1 dystonia knock-in mice breedings [13] were genotyped using a rapid-genotyping protocol (EZ Fast Tissue/Tail PCR Genotyping Kit, EZ BioResearch LLC, St. Louis, MO) [31,56,57]. 

### Culture

Neurons from individual newborn pups were cultured separately. Primary striatal neurons were cultured by a method described previously [31,56,57], with slight modifications. In brief, the striatum and the CA3-CA1 region of hippocampus were dissected at postnatal day 0–1, trypsinized, and mechanically dissociated. The cells were plated on 12-mm coverslips (thickness No. 0, Carolina Biological Supply, Burlington, NC) that were previously seeded with a rat glial feeder layer [58] in 24-well plates at a density of 24,000 cells per well. The feeder layer was seeded in the following plating medium: MEM (Invitrogen, Carlsbad, CA) containing 5 g/L glucose, 0.2 g/L NaHCO_3_, 100 mg/L bovine transferrin (EMD Chemicals, Gibbstown, NJ), 2 mM GlutaMAX (Invitrogen), 25 mg/L insulin, and 10% fetal bovine serum (FBS, Invitrogen). The feeder layers were then maintained in a 1:1 mixture of plating medium and growth medium. The growth medium contained the following: MEM containing 4 µM cytosine β-D-arabinofuranoside, 0.5 mM GlutaMAX, NS21 [59], and 5% FBS. The cells were cultured in a humidified incubator at 37°C with 5% CO_2_. The cultured striatal neurons were analyzed after 15-18 days in culture. The experimental data were obtained from 3-7 separate culture batches (i.e., pups) for each genotype (i.e., wild-type and heterozygous littermates). 

### Fluorescence imaging system for [Ca^2+^]_c_ experiments

Cells were imaged using an inverted microscope (Eclipse-TiE, Nikon, Melville, NY) that was equipped with a back-illuminated electron-multiplying charge-coupled device (EMCCD) camera (128 x 128 pixels, iXon^EM^+ DU-860, Andor Technology, Belfast, UK). The camera was perfused continuously with chilled water (Oasis 160 liquid recirculating chiller, Solid State Cooling Systems, Wappingers Falls, NY) to maintain a temperature of -80°C, thereby minimizing noise. 

The Ca^2+^ dye was excited using a 490-nm light-emitting diode (LED, CoolLED-Custom Interconnect, Hampshire, UK) at 10% of maximum intensity and imaged using an objective lens (Plan Fluor, 40x, NA1.30, Nikon), a filter cube (490/20-nm excitation, 510-nm dichroic long-pass and 520-nm long-pass emission), and no intermediate coupler (i.e., 1x). Images (14-bit) were acquired at 100 frames/sec with a 9.67-msec exposure, a pre-amplifier gain of 4.9, an EM gain of 50, a 10-MHz pixel readout rate, and 1x1 binning (Solis software, Andor Technology). Images in a stack were stored in TIFF format. Exposure of the neurons to the excitation light was minimized by turning on the LED only during image capture (triggering was timed using the digital output from the camera). 

For DIC imaging, the same objective lens and EMCCD camera were used with 1x1 binning, but the exposure time was 20 msec and no EM gain was used. 

### Perfusion

The reagents were applied to live neurons in the imaging chamber using the "Y-tube" method, a rapid local perfusion system that allows exchange of the external solution within 30 msec with an average travel rate of ~100 μm/msec near the cell [60-62]. This configuration ensures that the entire surface of the neuron is exposed continuously to fresh solution. The Y-tube was connected to a suction bottle with a vacuum level of 300-400 mmHg, and the system was controlled by a Master-8 pulse stimulator (AMPI, Jerusalem, Israel). In addition, reagent-free solution was continuously applied through the bath perfusion system. 

### Fluorescence imaging of [Ca^2+^]_c_

Neurons were loaded with the [Ca^2+^]_c_ indicator Fluo-5F-AM (Invitrogen) by immersing the cells in a 1-μM solution in MEM for 10 min in a culture incubator at 37°C [57]. After loading, the neurons were rinsed by transferring them to dye-free Tyrode’s solution (containing, in mM: 125 NaCl, 2 KCl, 2 CaCl_2_, 2 MgCl_2_, 30 glucose, 25 HEPES, pH 7.4, 310 mOsm) for 10 min at room temperature. The neurons were then transferred to an imaging chamber that was equipped with stimulation electrodes (RC-21BRFS, Warner Instruments, Hamden, CT). The neurons were washed with Tyrode’s solution using both the local and bath perfusion systems for 5-10 min, during which neurons were selected for imaging. 

Any biased acquisition of fluorescence images was avoided by preselecting neurons for imaging based on DIC optics (i.e., in the absence of prior information regarding the cell’s fluorescence status). The neurons were selected based on the following indicators of good health: a clear cellular margin, extended dendrites, a uniform glial layer underneath the neuron, a lack of clustered somata, and a lack of bundled neurites. In addition, the isolated neurons were mostly used for [Ca^2+^]_c_ imaging for morphological identification and for imaging of their dendrites (see Figure S1 in File S1). Non-isolated neurons that were in a cluster of cells tended to be small (i.e., a somatic area typically <158 μm^2^; Figure 5A). For each image, the neuron’s soma was positioned near the center of the visual field.

[Ca^2+^]_c_ transients were induced by applying 10 1-sec 30-mA field stimulation pulses at 1 Hz using a Master-8 pulse generator (AMPI) and an isolated stimulator (DS3, Digitimer, Hertfordshire, UK). The timing of the field stimuli was confirmed in a separate experiment in which illumination was triggered using the same pulse that controls the field stimulation and then measuring fluorescence intensity [63]. Neurons were imaged for a total of 30 sec (i.e., 10 sec before and 10 sec after stimulation in addition to the 10-sec stimulation period). After each experiment, 20 images were acquired under the same imaging conditions, but with the camera shutter closed, in order to measure the background intensity level. All imaging experiments were performed at room temperature.

Tetrodotoxin (TTX, 1 μM, Tocris Bioscience, Ellisville, MO) and cadmium (CdCl_2_, 200 μM) were dissolved in Tyrode’s solution and applied to the neurons through the Y-tube (the bath perfusion contained TTX- and cadmium-free Tyrode’s solution). The blockers were applied for 2 min before the start of imaging.

To prepare the Tyrode’s solutions with different extracellular Ca^2+^ concentrations ([Ca^2+^]_o_), the Mg^2+^ concentration ([Mg^2+^]_o_) was adjusted accordingly to maintain a fixed concentration of divalent cations such that [Ca^2+^]_o_ + [Mg^2+^]_o_ = 4 mM. The Ca^2+^-free solution was prepared by adding ethylene glycol-bis(2-aminoethylether)-*N,N,N*’,N’-tetraacetic acid (EGTA, 1 mM) to a solution containing 0 mM Ca^2+^ and 4 mM Mg^2+^ (pH 7.4, 310 mOsm). The Ca^2+^-free solution and the various [Ca^2+^]_o_ solutions were applied through both the Y-tube and bath perfusion for 5 min prior to the start of imaging.

### Image analysis for [Ca^2+^]_c_ experiments

The 14-bit images in a stack were converted to 16-bit images in a stack by opening the images in ImageJ (v1.46r, W. S. Rasband, NIH, available at http://imagej.nih.gov/ij/), and the Ca^2+^ signal was quantified using ImageJ and the relevant plug-ins. The shutter-closed (i.e., background noise) images were averaged and subtracted from all of the images in the stack for each experiment. After converting to 32-bit images, the images taken during 1 sec immediately before the stimulus were averaged to determine the pre-stimulus baseline fluorescence intensity (F_0_). The change in [Ca^2+^]_c_ was expressed as the fold change in fluorescence intensity (F) relative to baseline (F_0_) as follows: (F–F_0_)/F_0_=ΔF/F_0_ (using the function “Process/Image calculator”). Because no decline in baseline was observed during the 10-sec pre-stimulus period, the data were not corrected for photobleaching. The analyzed images were saved in TIFF format. 

### Intensity analysis of [Ca^2+^]_c_ in dendritic regions-of-interest (ROIs)

In each calculated image stack, the ΔF/F_0_ values in the defined ROIs were measured using ImageJ’s Time Series Analyzer V2.0 (courtesy of Dr. Balaji Jayaprakash). Three ROIs (each containing 2x2 pixels, 1.18 μm/pixel) were defined on each dendritic shaft using the DIC image as a guide with the investigator blinded to the fluorescence information. The ROIs were contiguous, and the most proximal pixel was 6 pixels (~7 μm) from the soma-dendrite border. Two or three dendrites were analyzed per neuron. A dendrite was not analyzed if the ROI exceeded the width of the dendrite identified by the DIC image or if the dendrite was equal to or shorter than 12 pixels long in the image. The ROIs were not placed at major dendritic branch points or where dendritic intersections with other neurites were visible. 

The time sequence of the ΔF/F_0_ values from each ROI was exported to Excel (Microsoft, Redmond, WA). The values of three ROIs in one dendrite were averaged to yield one trace. This average trace was then exported in text file format to the Mini Analysis Program (version 6.0.7, Synaptosoft, Fort Lee, NJ), converted to an Axon Binary File (ABF), and Gaussian-filtered at 15 Hz with respect to time. Two different types of analyses were then performed.

Experimental group 1 (Figures 1, 4, and 6): The [Ca^2+^]_c_ transients in responders were analyzed for peak amplitude and decay time constant using the Mini Analysis Program. The software uses the root-mean square (RMS) noise levels to detect the peak. RMS is measured between -1000 and -104 msec relative to the first stimulus (i.e., 0 msec is the time of the 1^st^ stimulus, 3.5 blocks, 1 block = 256 msec). A peak was detected if the intensity crossed the threshold of (RMS x 5). The amplitude of the detected peak was then measured relative to the baseline level. The decay time constants were measured from 80% to 20% of the peak amplitude based on the following equation: F(t) = a*exp(-t/τ), where F(t) is the fluorescence intensity at time t during the decay, a is the amplitude, and τ is the decay time constant. The fitting was performed without applying any constraint. For the fitting analysis, the Gaussian filter was changed to 5 Hz to reduce noise. The decay time constants were discarded if the curve-fitting yielded a fit with r^2^<0.990, as these infrequent traces typically contained high noise levels. Each trace was examined visually to confirm the software analysis contained no errors. Cumulative [Ca^2+^]_c_ change was calculated as the integral of the ΔF/F_0_ trace starting from the stimulation onset until 1 sec after each pulse (with Excel). The calculation was based on the absolute values of ΔF/F_0_, without any normalization.

Experimental group 2 (Figures 2 and 3): In some traces, a [Ca^2+^]_c_ transient could not be detected. In such experiments, only the peak amplitude and F_0_ were analyzed (using Excel). The peak of the [Ca^2+^]_c_ elevation was calculated as the highest point 10-70 msec after the stimulus, and the amplitude was measured as the difference between this peak and F_0_.

### Intensity analysis [Ca^2+^]_c_ along a series of single pixels

Using the DIC image, a series of contiguous pixels was assigned along the middle of a dendritic shaft, starting from approximately the center of nucleus, moving through the somatic cytoplasm to the distal dendrite (Figure 3). Changes in ΔF/F_0_ at individual pixels were analyzed as described above for experimental group 2. 

### Impact of changes in decay time constants

A change in the time constant of exponential decay can be used to estimate the impact of a change in [Ca^2+^]_c_ with respect to at least two basic features of [Ca^2+^]_c_ dynamics. First, the area under a [Ca^2+^]_c_ transient corresponds to the total [Ca^2+^]_c_ load. After peak amplitude was normalized to 1, the fluorescence intensity at time t during the decay (F(t)) was expressed as follows: F(t) = exp(-t/τ). The integral of F(t) from the time of stimulation (t = 0) to time t is then: Area(t) = τ*(1-exp(-t/τ)). When t approaches infinity, Area(∞) = τ. Thus, the ratio of the total areas under different conditions is expressed as the ratio of the τ’s. Second, the time T during which [Ca^2+^]_c_ remains above a specified threshold value has the following relationship: threshold = exp(-T/τ). Therefore, T = - τ*ln(threshold) = τ*ln(1/threshold). Thus, the ratio of these parameters under various conditions is given by the ratio of the τ’s. These simple calculations illustrate that a change in the decay time constant can be used to estimate the impact of changing [Ca^2+^]_c_.

### Immunocytochemistry

Immunocytochemistry was performed as described previously [31]. In brief, the cultured neurons were fixed, blocked and permeabilized. Thereafter, the neurons were treated overnight (15–21 h) at 4°C with one or more of the following primary antibodies: rabbit polyclonal anti-DARPP-32 (AB10518, EMD Millipore, Billerica, MA) (400x dilution), mouse monoclonal anti-GAD65 (MAB351, EMD Millipore) (1000x dilution), mouse monoclonal anti-VGAT (131 011, Synaptic Systems, Göttingen, Germany) (1000x dilution), rabbit polyclonal anti-MAP2 (AB5622, EMD Millipore) (400x dilution), rabbit polyclonal anti-VAChT (139 103, Synaptic Systems) (400x dilution), and/or guinea-pig polyclonal anti-VGLUT1 antibodies (AB5905, EMD Millipore) (1000x dilution). After incubation in the primary antibody, the neurons were washed, then incubated for 60 min at room temperature with the appropriate secondary antibodies as follows: goat anti-mouse antibody conjugated with Alexa Fluor 488 (A-11001, Invitrogen) (1000x dilution), goat anti-rabbit IgG antibody conjugated with Alexa Fluor 405 (A-31556, Invitrogen) (1000x dilution), or goat anti-guinea pig IgG antibody conjugated with Alexa Fluor 594 (A-11076, Invitrogen) (1000x dilution). 

### Imaging and analysis of immunocytochemical data

Cells were imaged using an interline CCD camera (Clara, Andor Technology). The camera was cooled to -45°C aided by an internal fan. Alexa Fluor 594 was excited using a 595-nm LED (CoolLED-Custom Interconnect) at 100% intensity and imaged with a filter cube (590/55-nm excitation, 625-nm dichroic long-pass, 665/65-nm emission) using a 5-sec exposure. Alexa Fluor 488 was excited using a 465-nm LED (CoolLED-Custom Interconnect) at 100% intensity and imaged with a filter cube (490/20-nm excitation, 510-nm dichroic long-pass, 530/40-nm emission) using a 5-sec exposure. Alexa Fluor 405 was excited using a 400-nm LED (CoolLED-Custom Interconnect) at 100% intensity and imaged with a filter cube (405/40-nm excitation, 440-nm dichroic long-pass, 470/40-nm emission) using a 5-sec exposure. Images (16-bit) were acquired using an objective lens (Plan Fluor, 40x) without a coupler or binning using the single-image capture mode of the Solis software (Andor Technology). Using phase-contrast optics, the fields of view were scanned systemically without bias with respect to the cell type, i.e. images of small cells were acquired in contrast to [Ca^2+^]_c_ imaging. 

Somatic area was measured from an image captured using transmitted light optics (either DIC or phase-contrast) by manually tracing the perimeter with ImageJ. Somatic fluorescence intensity was measured as the peak value of the intensity profile along a line drawn through the center of the soma, after the profile was filtered using a running average of 7 pixels.

For establishing an intensity threshold for cellular classification, we analyzed the fluorescence intensity of a negative control in which the primary antibody was omitted from the immunocytochemical procedure. After the x-values (somatic areas) were transformed using a base-10 logarithmic function, we applied a linear regression line and analyzed the 95% uniform confidence band [64]. Regardless of the somatic area, this band will cover the mean intensity with 95% of probability. The upper limit of the confidence band was used as the intensity threshold to determine whether the cell was positive or negative for a given antigen.

### Drugs

All chemical reagents were purchased from Sigma-Aldrich (St. Louis, MO) unless otherwise specified. The stock solutions were prepared as follows: TTX was dissolved in distilled water at 1 mM, and CdCl_2_ was dissolved in distilled water at 100 mM. The reagents were diluted to their final working concentration in Tyrode’s solution.

### Statistical analyses

Summary data are presented as mean ± SEM. Statistical significance was determined using the Student's *t*-test with two-tailed p-values after data were log-transformed. 

## Supporting Information

File S1 **Supporting figures. Figure S1**, Montage of phase-contrast images illustrates a variety of striatal neuron types in dissociated culture. In both wild-type and heterozygous cultures, large cells were relatively isolated (arrows), and small cells were clustered (arrowheads). **Figure S2**, Some neurons failed to respond to a stimulus train. (A) DIC images of a wild-type (somatic area, 220 μm2) and heterozygous (somatic area, 362 μm2) neuron. The ROIs used to measure the data shown in panel B are indicated. (B) ΔF/F0 was measured at the indicated ROIs during a 10-pulse stimulation train at 1 Hz. **Figure S3**, Neither wild-type nor heterozygous neurons showed a correlation between dendrite width and somatic area (r=0.324, p=0.062 for wild-type and r=0.185, p=0.295 for heterozygous neurons).
(DOC)
