# Supplementary material for: Minimal Change in the Cytoplasmic Calcium Dynamics in Striatal GABAergic Neurons of a DYT1 Dystonia Knock-In Mouse Model
Source: PLoS One. 2013 Nov 19;8(11):e80793. doi: 10.1371/journal.pone.0080793 (PMC3834333; doi:10.1371/journal.pone.0080793)
Supplement: File S1 — Supporting figures. Figure S1, Montage of phase-contrast images illustrates a variety of striatal neuron types in dissociated culture. In both wild-type and heterozygous cultures, large cells were relatively isolated (arrows), and small cells were clustered (arrowheads). Figure S2, Some neurons failed to respond to a stimulus train. (A) DIC images of a wild-type (somatic area, 220 μm2) and heterozygous (somatic area, 362 μm2) neuron. The ROIs used to measure the data shown in panel B are indicated. (B) ΔF/F0 was measured at the indicated ROIs during a 10-pulse stimulation train at 1 Hz. Figure S3, Neither wild-type nor heterozygous neurons showed a correlation between dendrite width and somatic area (r=0.324, p=0.062 for wild-type and r=0.185, p=0.295 for heterozygous neurons). (DOC) [file pone.0080793.s001.doc]

**SUPPORTING INFORMATION (by Iwabuchi, Koh, Wang, Ho and Harata)**

**
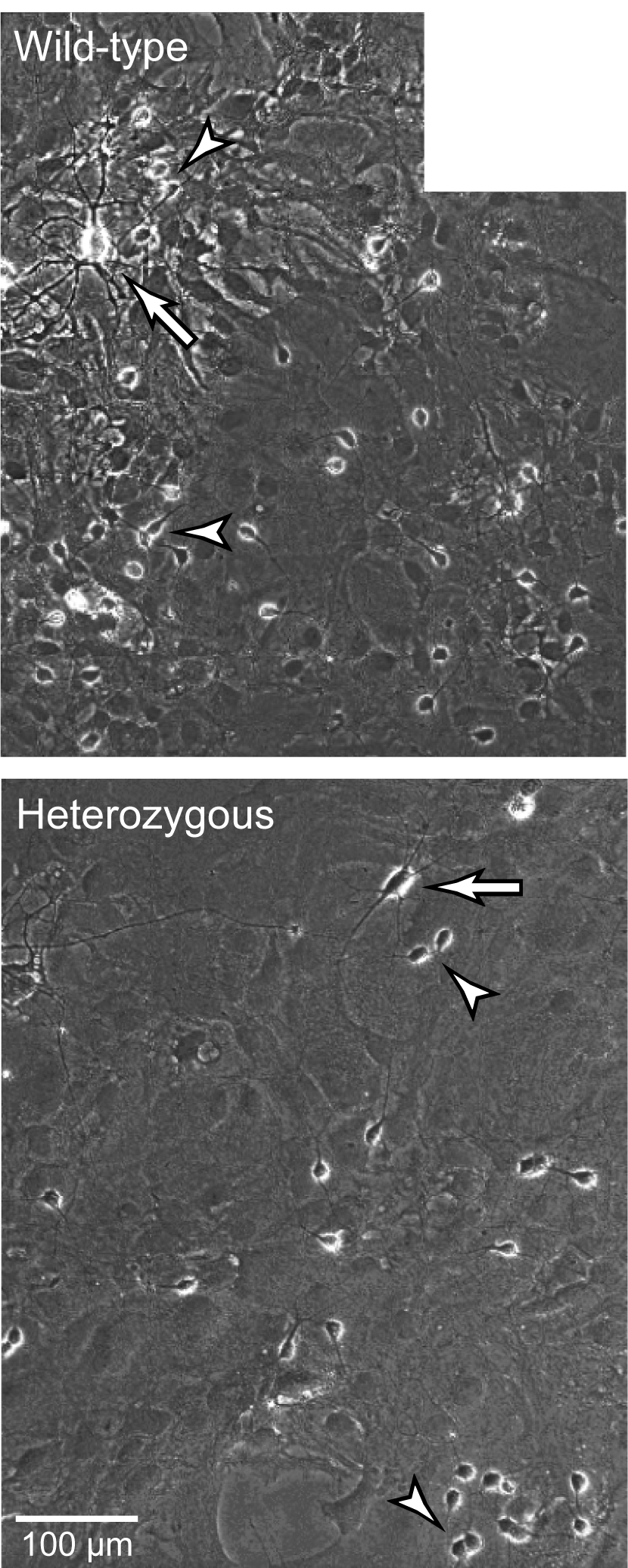
**

**Figure S1**. Montage of phase-contrast images illustrates a variety of striatal neuron types in dissociated culture. In both wild-type and heterozygous cultures, large cells were relatively isolated (arrows), and small cells were clustered (arrowheads).


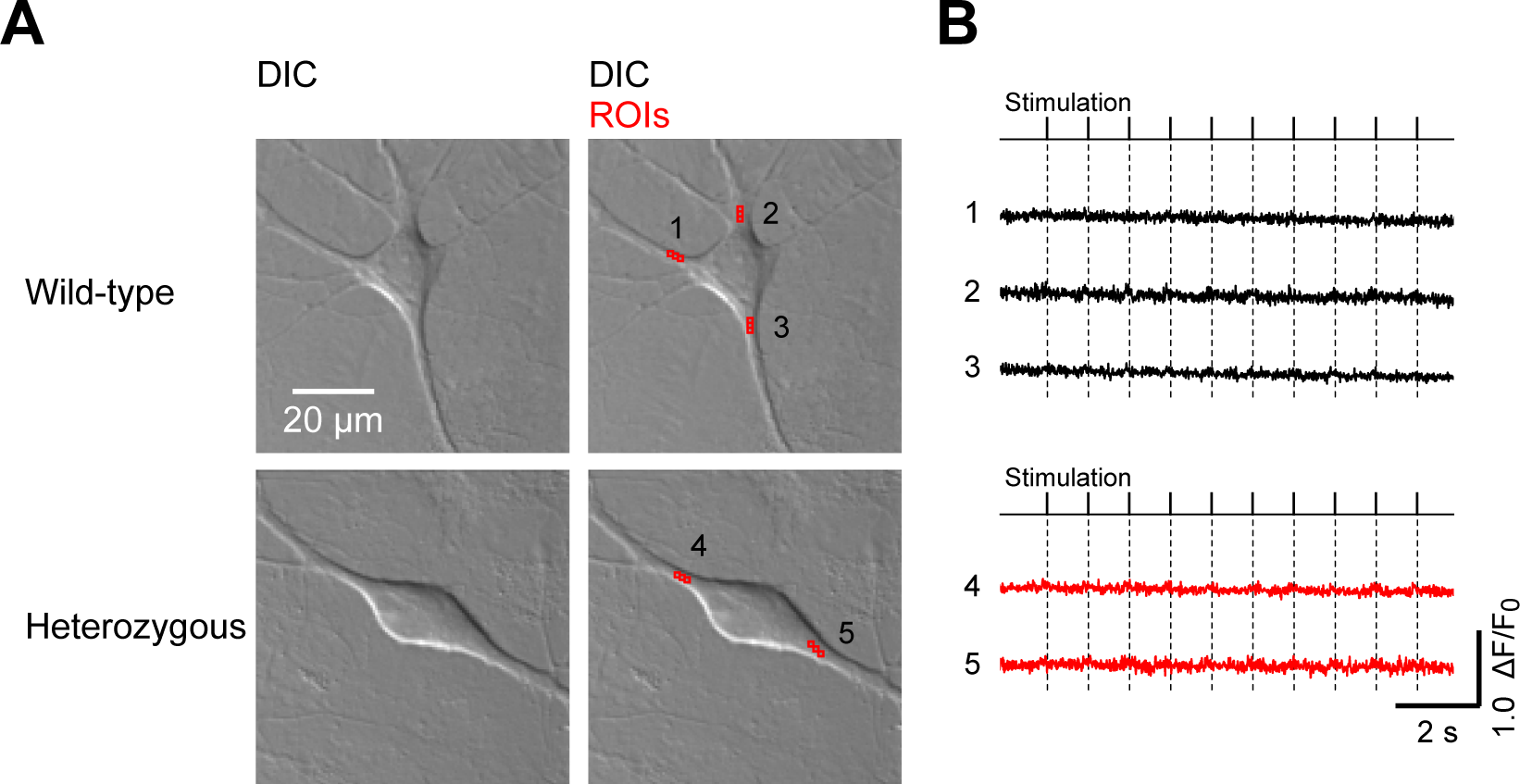


**Figure S2**. Some neurons failed to respond to a stimulus train. (A) DIC images of a wild-type (somatic area, 220 μm^2^) and heterozygous (somatic area, 362 μm^2^) neuron. The ROIs used to measure the data shown in panel B are indicated. (B) ΔF/F_0_ was measured at the indicated ROIs during a 10-pulse stimulation train at 1 Hz.


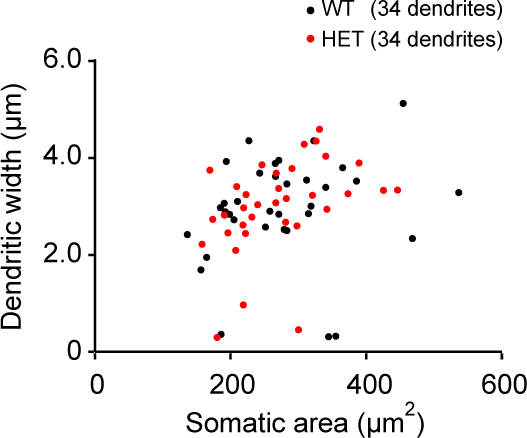


**Figure S3**. Neither wild-type nor heterozygous neurons showed a correlation between dendrite width and somatic area (r=0.324, p=0.062 for wild-type and r=0.185, p=0.295 for heterozygous neurons).
